# The Green Scalpel: Aligning United Arab Emirates Perioperative Care With the National Net Zero 2050 Mandate

**DOI:** 10.7759/cureus.106132

**Published:** 2026-03-30

**Authors:** Noun Gadalla, Amr Fahim

**Affiliations:** 1 Trauma and Orthopedic, Rashid Hospital, Dubai, ARE; 2 Plastic and Reconstructive Surgery, Rashid Hospital, Dubai, ARE

**Keywords:** anesthetic gas emission, green surgery, healthcare waste management, operating room carbon footprint, perioperative sustainability, uae net zero 2050

## Abstract

Climate change threatens global health, yet the healthcare sector itself is a significant contributor to the problem. Operating rooms (ORs) are the most resource-intensive areas within hospitals, consuming substantial energy, generating considerable waste, and emitting volatile anesthetic gases. While the United Arab Emirates (UAE) has committed to achieving Net Zero by 2050, perioperative sustainability strategies remain absent from national healthcare policy-a critical gap in the country's decarbonization efforts.

This literature review synthesizes findings from 37 peer-reviewed publications, institutional sustainability reports, and policy documents to examine operating room environmental impacts, waste management practices, global sustainability interventions, and UAE-specific healthcare initiatives. Sources were analyzed to extract data on waste generation, energy consumption, anesthetic gas emissions, international best practices, and regional sustainability programs.

Global evidence shows that operating rooms contribute to healthcare emissions through three main pathways: waste generation, energy consumption, and anesthetic gas emissions. Multidisciplinary Green Teams implementing the 5R circular economy framework (Reduce, Reuse, Recycle, Rethink, Research) have successfully reduced environmental impacts while maintaining clinical safety standards. Effective interventions include comprehensive waste segregation programs, transitioning to reusable medical equipment, investing in energy-efficient technologies, and optimizing anesthetic gas delivery systems.

While the UAE has made progress in broader sustainability practices, significant gaps exist in OR-specific data and implementation guidelines. Substantial perioperative emission reductions are achievable through systematic implementation of evidence-based interventions. However, this requires comprehensive audits, targeted policy development, and stakeholder engagement to align the UAE's perioperative care with its Net Zero 2050 commitment.

## Introduction and background

Climate change represents a severe threat to global health [1]. Ironically, the healthcare sector substantially contributes to environmental harm, responsible for roughly 4.6% of worldwide greenhouse gas (GHG) emissions and producing considerable institutional waste [2,3]. Surgical theaters are particularly resource-intensive, using three to six times more energy than other hospital areas and accounting for 20-33% of overall hospital waste [4,5]. With more than 300 million surgical operations conducted globally each year, perioperative services constitute a major contributor to healthcare-associated emissions [3].

The United Arab Emirates (UAE) has shown environmental commitment with its net-zero emissions pledge by 2050--becoming the first Middle East and North Africa (MENA) country to set this goal. Despite healthcare's significant environmental impact, perioperative services have not been explicitly included in national sustainability strategies, highlighting a notable gap in comprehensive decarbonization efforts [6].

Reducing carbon emissions in surgical environments necessitates targeting three main "carbon hotspots": energy-inefficient infrastructure, procurement choices, and anesthetic gas management. Volatile anesthetic gases-notably desflurane, with a global warming potential 2,540 times that of CO₂-are significant sources of emissions [5]. Heating, ventilation, and air conditioning (HVAC) systems account for over 90% of operating theater energy use, with one UK operating room (OR) consuming enough electricity to supply more than 2,000 households annually [3]. One surgical operation generates more waste than a four-person household produces in a week, with up to 90% of hazardous waste incorrectly segregated and incinerated, resulting in disposal expenses 10-20 times higher than for non-hazardous waste [7].

Global evidence, including the NHS Greener NHS initiative, shows that circular economy strategies--the 5R framework (Reduce, Reuse, Recycle, Rethink, Research)--deliver environmental, financial, and clinical advantages [8]. The NHS targets net-zero emissions by 2045, while comparable initiatives in North America, Europe, and Australia have achieved measurable reductions in emissions, waste, and costs while preserving or enhancing patient safety [9-11].

The UAE has launched encouraging sustainability initiatives, including the Emirates Health Services (EHS) Green Patient Care program, which enabled 300,000 electronic consultations in 2023, allegedly decreasing carbon emissions by over six million tonnes [9]. Sheikh Shakhbout Medical City (SSMC) has stressed the need to expand sustainability beyond environmentally friendly buildings to include safer systems and supply chain oversight [10].

Nevertheless, considerable challenges remain in medical waste management. Evaluations show substantial shortcomings in waste segregation, handling procedures, and personnel training, with numerous institutions missing comprehensive waste management policies [12]. Recent assessments of Dubai Health Authority facilities have identified ongoing barriers that require standardized frameworks and strengthened regulatory supervision [13]. Existing initiatives remain disjointed and lack systematic, evidence-based approaches necessary to align perioperative services with the National Net Zero 2050 mandate.

This review synthesizes international evidence on the environmental impact of operating theaters, assesses validated sustainability interventions, and analyzes the UAE healthcare landscape to recommend a strategic framework that aligns perioperative services with national climate objectives. By combining international best practices with regional priorities, this manuscript offers actionable guidance for healthcare administrators, policymakers, and clinical teams pursuing high-quality surgical care while promoting environmental stewardship.

## Review

Methodology 

This review was conducted in accordance with the Preferred Reporting Items for Systematic Reviews and Meta-Analyses (PRISMA) 2020 guidelines to ensure transparency and reproducibility [14]. A comprehensive literature search was performed in PubMed/MEDLINE and Scopus databases using keywords including "sustainable healthcare," "green surgery," "environmental sustainability," "operating room," "perioperative care," "waste management," "energy efficiency," "anesthetic gases," "carbon footprint," and "UAE healthcare." The search covered publications from January 2008 to December 2025, limited to English-language articles.

Studies were included if they reported sustainability initiatives in healthcare or surgical settings, including waste reduction, energy efficiency, anesthetic gas management, water conservation, or sustainable supply chain practices. Eligible study designs included peer-reviewed research articles, systematic reviews, case studies, and quality improvement reports that provided empirical data on environmental impact, cost savings, or implementation feasibility. Studies were excluded if they were not directly related to healthcare sustainability, lacked sufficient quantitative or qualitative data, were published before 2008, or were not available in English.

The initial database search identified 260 records (PubMed: 142, Scopus: 118). After removing 85 duplicates, 175 unique records underwent title and abstract screening by two independent reviewers. Records that did not meet the inclusion criteria were excluded at this stage (n = 118), leaving 57 full-text articles for detailed assessment. Following full-text review, 26 additional articles were excluded due to insufficient quantitative data or failure to meet all inclusion criteria (12 lacked quantitative data, six were wrong publication types, five lacked direct focus, and three were not in English). An additional six records were identified through website and organizational searches and were all included after assessment. 

**Figure 1:**
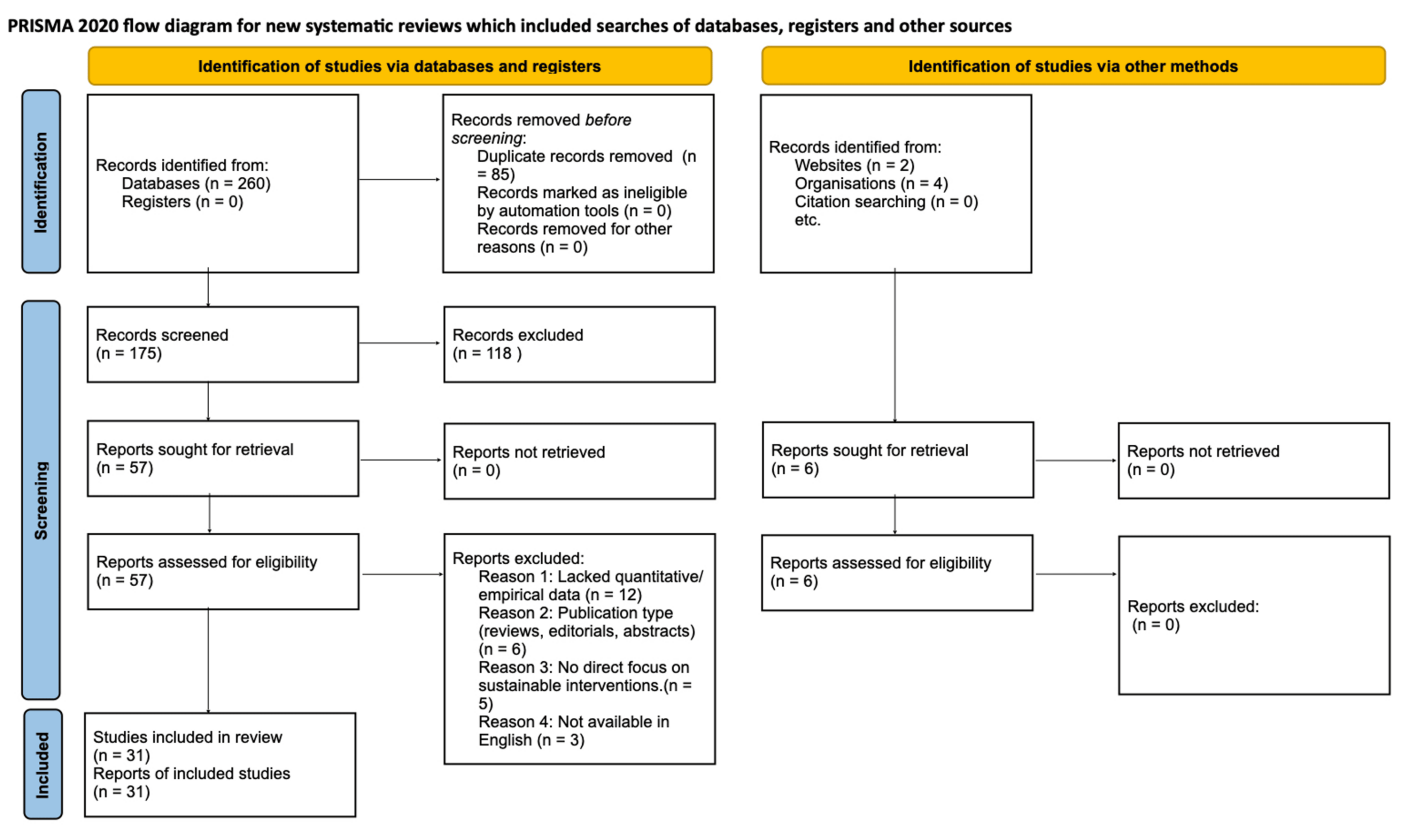
PRISMA 2020 flow diagram. PRISMA: Preferred Reporting Items for Systematic Reviews and Meta-Analyses.

Data were systematically extracted using standardized forms to capture study characteristics, intervention types, implementation strategies, outcomes, and relevance to the UAE context. Study quality was assessed using appropriate critical appraisal tools for the study design: A Measurement Tool to Assess Systematic Reviews (AMSTAR-2) for systematic reviews and the Standards for Quality Improvement Reporting Excellence (SQUIRE) guidelines for quality improvement studies. Given the heterogeneity of study designs and interventions, a narrative synthesis approach was employed, with studies organized thematically by intervention type (waste management, energy efficiency, anesthetic gases, water conservation, and supply chain optimization). The study selection process is illustrated in Figure 1.

Results

This review of 37 publications revealed that operating rooms disproportionately contribute to healthcare's environmental footprint, generating 20-33% of hospital waste, using three to six times more energy than other departments, and emitting significant anesthetic gases, with desflurane having a global warming potential 2,540 times that of CO₂ [3,5]. A single surgical procedure produces about 173 kg CO₂e and generates more waste than a family of four in one week, with 43.9% of this waste potentially recyclable [3,6,15].

UAE-specific data showed varied medical waste generation rates across UAE facilities (0.3-1.95 kg/bed/day), and assessments revealed inconsistent waste-tracking systems and limited waste-minimization practices in surveyed facilities [12,13]. These findings emphasize the urgent need for comprehensive green surgery initiatives to reduce the environmental impact of perioperative care in the UAE, aligning with international best practices for sustainable healthcare [15]. Progress was evident in broader sustainability efforts, such as the Green Patient Care project, which facilitated 300,000 electronic visits and reduced CO₂ emissions by more than six million tons in 2023 [9]. However, critical gaps appeared in operating room-specific sustainability data, measurement standards, and the integration of perioperative sustainability into national healthcare policies.

Global evidence demonstrated that multidisciplinary Green Teams implementing the 5R framework (Reduce, Reuse, Recycle, Rethink, Research) achieved measurable environmental and economic benefits, including 50% waste volume reductions, 50-75% decreases in anesthetic agent consumption through low-flow techniques, and substantial cost savings ranging from $26,000 to $1.77 million annually across different interventions [16].

Environmental footprint of operating rooms

**Figure 2:**
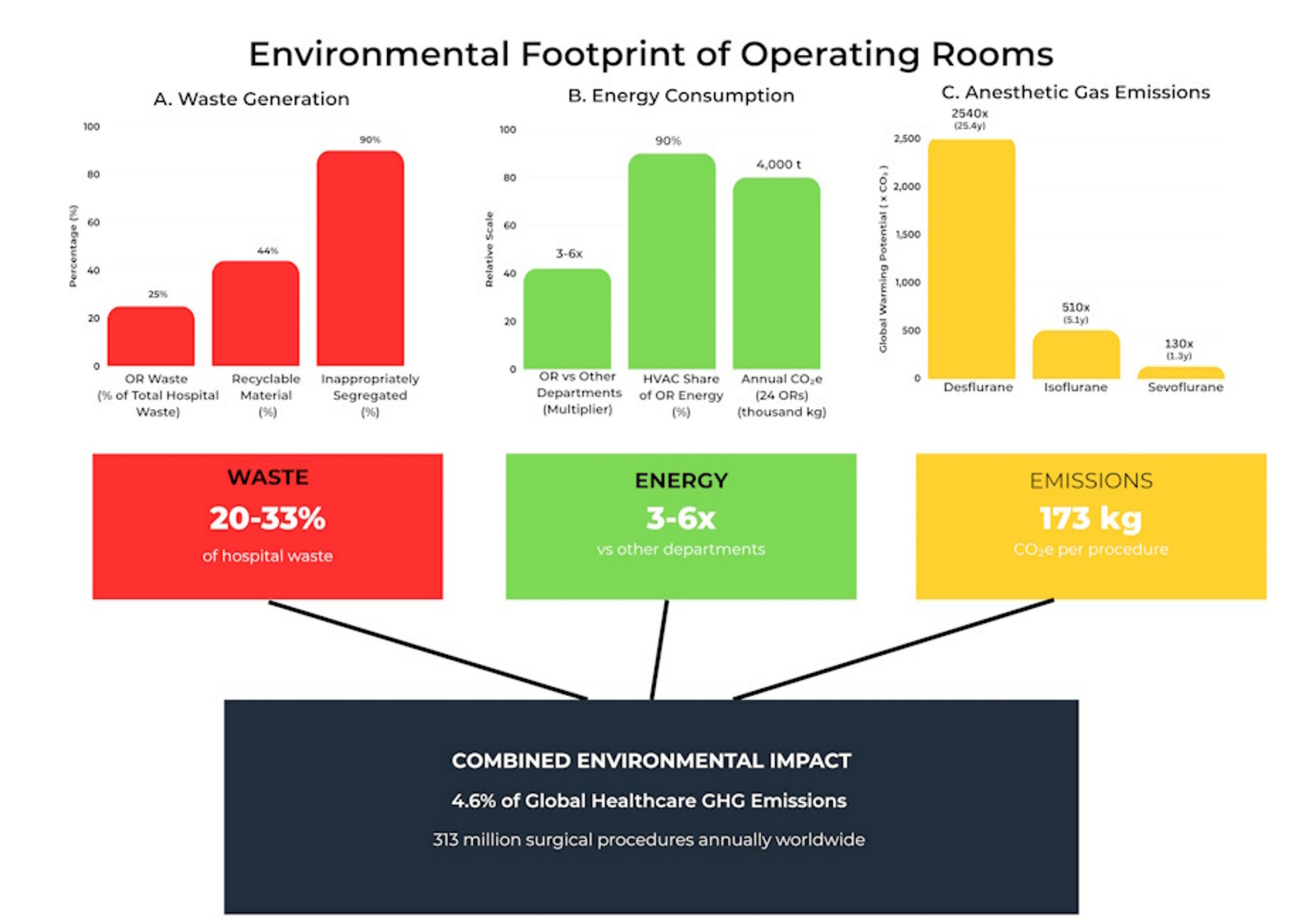
Environmental footprint of operating rooms. This figure illustrates the three major domains of environmental impact from operating rooms (ORs): (A) waste generation showing that ORs generate 20-33% of hospital waste with 44% recyclable material but 90% inappropriately segregated; (B) energy consumption demonstrating that ORs consume three to six times more energy than other departments with heating, ventilation, and air conditioning (HVAC) systems accounting for 90% of usage; (C) anesthetic gas emissions displaying the global warming potential of different agents (desflurane 2,540×, isoflurane 510×, sevoflurane 130× compared to CO₂). The bottom panel shows the combined environmental impact contributing to 4.6% of global healthcare greenhouse gas emissions from 313 million annual surgical procedures worldwide. Source: Created by authors based on data synthesized from references [1-3,5-8,12,13]. Artwork created using CANVA Image Editor (Sydney, Australia).

Operating rooms are among the most resource-intensive environments within healthcare facilities, generating substantial environmental impacts across multiple domains (Figure 2). Understanding the scale and sources of these impacts is essential for developing targeted interventions.

Waste Generation and Composition

Surgical waste presents a significant environmental and economic challenge. Operating rooms generate 20-33% of total hospital waste, with a single surgical procedure producing more waste than a family of four in a week [3]. In the United States, healthcare facilities produce over 6,600 tons of waste daily, more than four billion pounds annually, with operating rooms and labor-delivery units accounting for about 70% of this volume [8]. The composition of surgical waste highlights considerable potential for improvement. Approximately 43.9% of waste generated per surgical procedure is potentially recyclable, with large joint arthroplasties (total hip and knee replacements) producing the highest volumes of recyclable materials (12.6 kg and 13.1 kg per case, respectively) [15]. Evidence showed that 80% of this waste is generated before the first incision [3]. Moreover, 90% of hazardous waste is improperly segregated and unnecessarily incinerated, resulting in environmental pollution, soil and water acidification, and mercury contamination [3]. Furthermore, incineration costs 10-20 times more than non-hazardous waste disposal, adding a substantial financial burden to the environmental harm [3].

Packaging materials are a major contributor to surgical waste, accounting for up to 40% of regulated operating room waste [7]. Blue wrap, used to store sterile instruments, accounts for about 19% of operating costs [3]. Importantly, up to 80% of perioperative waste is generated before the patient enters the operating room, suggesting that pre-incision segregation could significantly reduce biohazardous waste volume [3].

In the UAE, medical waste generation rates vary considerably between facilities. A 2008 study reported an average of 1.95 kg of medical waste per bed per day across 14 hospitals, with total medical waste in UAE hospitals estimated at 5,600 tons annually in 2001 [12]. More recent data from Dubai Health Authority facilities indicate lower rates of 0.28-0.33 kg per bed per day, with an average of 0.3 kg per bed per day [13]. These variations likely reflect differences in waste management practices, facility types, and the use of disposable items. Notably, many UAE hospitals lack waste-minimization measures, such as centralized purchasing and the selection of less-wasteful materials, and some facilities lack pretreatment capabilities for medical waste, potentially due to budgetary constraints [12,13].

Energy Consumption and Carbon Emissions

Operating rooms are extremely energy-intensive, consuming three to six times more energy than other hospital areas [14]. More than 90% of this energy use is attributed to HVAC systems and powered equipment needed to maintain sterile, temperature-controlled environments. In the United Kingdom, a high-volume center with 24 operating rooms generates over four million kg CO₂e annually from HVAC energy use alone. To put this in perspective, the energy required for a single operating room could power over 2,000 homes in the UK [15].

Beyond energy use, the environmental impact of these emissions has direct consequences for public health. In the United States, healthcare-related emissions contribute to 10% of photochemical smog and 9% of respiratory diseases, resulting in an estimated 405,000 disability-adjusted life-years (DALYs) lost annually [17]. Furthermore, the lifecycle of surgical equipment, such as dental burs, highlights that moving from disposable to reusable items can significantly lower the greenhouse gas footprint associated with surgical procurement [18].

The carbon footprint of surgical procedures varies by complexity and duration. In the UK, a typical operation generates around 173 kg CO₂e, with estimates ranging from 146 to 232 kg CO₂e per procedure in Canada, the United States, and the United Kingdom [18]. A typical operating department in a large UK hospital produces over 5,000 tons of CO₂e per year, equivalent to driving an average petrol car 580 times around the Earth [19].

Anesthetic Gas Emissions

Volatile anesthetic gases are a significant and often underrecognized source of healthcare carbon emissions. Desflurane, sevoflurane, and isoflurane--the most commonly used inhaled anesthetics--are potent greenhouse gases with global warming potentials much greater than CO₂. Desflurane's global warming potential is 2,540 times that of CO₂, while sevoflurane and isoflurane have global warming potentials of 130 and 510, respectively [5]. Depending on the agent and fresh gas flow rates used, anesthetic gases can contribute up to 88% of an operating room's carbon footprint [16].

The environmental impact of anesthetic gases goes beyond their global warming potential. These agents persist in the atmosphere for significant periods: desflurane for 14 years, sevoflurane for 1.1 years, and isoflurane for 3.2 years, contributing to long-term climate effects [5]. In 2013, US healthcare activities accounted for 9-10% of national greenhouse gas emissions, with anesthetic gases comprising a measurable component of this footprint [18].

Global data and evidence

The environmental footprint of the global healthcare sector has been extensively quantified, highlighting both the scale of the challenge and the potential for meaningful interventions. Healthcare is responsible for approximately 4.6% of global greenhouse gas emissions, with substantial variation across countries and health systems [1].

In addition to greenhouse gases, healthcare-related emissions contribute to acid rain (12% of the national total in the US), photochemical smog (10%), and respiratory disease (9%). Public health damages from exposure to non-GHG emissions are estimated at 405,000 disability-adjusted life-years (DALYs) annually in the United States, primarily due to particulate matter emissions-a burden comparable to that of preventable medical errors [17].

Surgical care is a major contributor to healthcare resource consumption. In the UK, the carbon footprint of surgical care was estimated at 5.7 million tons of CO₂e in 2019 [19]. Globally, an estimated 313 million surgical procedures were performed in 2012, marking a one-third increase in volume over eight years [19].

The NHS in England remains the only healthcare system with a published national strategy addressing climate change, generating approximately 25 megatons of CO₂e annually, accounting for 4-5% of the nation's total GHG emissions [6]. The Greener NHS program targets net-zero emissions by 2045, with interim milestones and comprehensive strategies spanning all healthcare domains [6].

Global best practices in perioperative sustainability

International experience shows that sustainable perioperative practices are achievable, cost-effective, and clinically safe. Evidence-based interventions encompass multiple domains, ranging from organizational structures to specific technical modifications.

Green Teams: Collaborative Implementation Framework

Green Teams serve as the foundational organizational structure for implementing sustainable perioperative practices [16]. These multidisciplinary, collaborative support networks include surgeons, anesthetists, scrub and circulating nurses, residents, clinical engineers, and administrative staff who work together to promote and implement environmentally responsible behaviors in the workplace. Green Teams offer hospital executives and clinicians measurable results through periodic reports on achieved targets, fostering accountability and driving continuous improvement [16].

The Green Team model has been successfully implemented in diverse healthcare settings worldwide. These teams act as change agents by identifying opportunities for improvement, piloting interventions, measuring outcomes, and disseminating best practices across their institutions. The collaborative approach ensures that sustainability initiatives are clinically appropriate, operationally feasible, and aligned with patient safety priorities [16].

A Green Team's agenda should be organized around clear priorities and measurable objectives. The team establishes baseline metrics for waste generation, energy consumption, procurement patterns, and carbon emissions, then implements targeted interventions with ongoing monitoring and reporting. Effective Green Teams engage frontline staff through education, provide visible progress updates, and celebrate achievements to sustain engagement and motivation [16].

The 5R Framework: Reduce, Reuse, Recycle, Rethink, Research

The 5R framework offers a systematic approach to sustainable perioperative practices, guiding Green Teams and individual practitioners in identifying and implementing effective interventions [16].

Reduce focuses on minimizing resource consumption and waste generation. Strategies include standardizing surgical packs to remove unnecessary items, adopting leaner instrument trays, and reducing packaging materials. One study found that customized lean surgical packs in hand surgery, combined with the Wide Awake Local Anesthesia No Tourniquet (WALANT) method, generated 12% less waste compared to traditional sedation and local anesthetic approaches [20,21].

Reuse emphasizes transitioning from single-use to reusable items where clinically appropriate and economically viable. Life cycle assessments consistently show that reusable surgical instruments, gowns, and drapes have lower environmental impacts than disposable alternatives. Systematic reviews of the economic impact of these transitions indicate that reprocessing single-use medical devices can result in significant cost reductions for healthcare facilities [22]. Furthermore, current evidence on the sustainability of perioperative textiles suggests that reusable options have a superior environmental profile throughout their life cycle compared to disposable equivalents [23]. Reusable surgical gowns reduced natural energy consumption by 64%, greenhouse gas emissions by 66%, and blue water consumption by 83% [24]. This transition aligns with the strategic objectives for Abu Dhabi's health system, which emphasize embedding environmental sustainability and operational resilience into long-term clinical planning [25]. One hospital reduced waste by 50,000 pounds and saved $60,000 annually by switching to reusable surgical gowns, while another reduced blue wrap usage by 70% by switching to hard instrument cases, with estimated yearly savings of $26,000 [23].

Recycle involves proper waste segregation to maximize recycling and minimize incineration of non-hazardous materials. Improved segregation in operating rooms has reduced inappropriate disposal and increased recycling rates [8]. One medical center implemented a simple system: using clear bags during surgical preparation and switching to red bags only when the patient entered the operating room, aligning with the period when most biohazardous waste is generated. This change, along with washing and reusing scrubs and jackets, resulted in a 50% reduction in medical waste volume over seven years [8].

Studies indicate that up to 43.9% of waste per surgical procedure is recyclable, with large joint arthroplasties producing the most recyclable waste [15]. Establishing intraoperative recycling bins or waste-sorting facilities encourages recycling, and hospitals can partner with local waste management companies to recycle less-common items such as blue wrap, metals, and glass [15].

Rethink challenges conventional practices and encourage innovation in surgical care delivery. This includes adopting minimally invasive techniques, using regional anesthesia instead of general anesthesia when appropriate, maximizing day-case surgery, and implementing digital health solutions. The Getting It Right First Time (GIRFT) program in orthopedic surgery in the UK improved efficiency, savings, and patient care, reducing 380,000 inpatient bed days, preventing 5,000 emergency readmissions, and avoiding 49,000 unnecessary procedures between 2014 and 2019, equating to a reduction of approximately 26.5 kt CO₂e [19].

Telemedicine offers a significant opportunity to reduce carbon emissions. Telephone consultations generated 5,846 kg CO₂e (58%) fewer emissions than face-to-face appointments, reducing travel-related emissions by 66% per patient [4]. Digitally enabled self-care also yields financial savings: after lower limb arthroplasty, a virtual rehabilitation program was associated with $2,745 in savings per patient compared with traditional care [15].

Research underscores the importance of ongoing investigation, measurement, and innovation. This includes conducting life cycle assessments of surgical products and practices, measuring institutional carbon footprints, evaluating sustainability interventions, and disseminating findings to advance the field. Research also includes education and training programs focused on proper waste segregation and the benefits of sustainability, which have been shown to reduce biohazardous waste and increase recycling rates [24].

Anesthetic Gas Reduction Strategies

Evidence-based strategies to reduce anesthetic gas environmental impact include:

Agent selection: Establishing standardized clinical guidelines is a primary step in reducing surgical emissions [26]. As the global healthcare sector remains a primary driver of greenhouse gas emissions, transitioning to low-carbon clinical pathways is a critical component of the urgent public health response to the climate crisis [27]. Preferentially use sevoflurane or isoflurane over desflurane for routine cases, reserving desflurane for situations where its pharmacokinetic properties offer clear clinical advantages [28].

Low-flow anesthesia techniques: Reduce fresh gas flows to ≤1 L/min during the maintenance phase. This can decrease anesthetic agent consumption by 50-75% while maintaining adequate anesthetic depth and patient safety [16].

Total intravenous anesthesia (TIVA): Use propofol-based TIVA as an alternative to volatile agents when appropriate. This eliminates anesthetic gas emissions entirely and may reduce postoperative nausea and vomiting [19].

The WALANT technique: Using the WALANT technique in hand surgery demonstrates the environmental and economic benefits of regional anesthesia [21]. Evidence from laparoscopic surgery suggests that such shifts in clinical practice, including optimizing surgical trays and reducing single-use items, are essential strategies for mitigating the high greenhouse gas emissions associated with traditional operating room environments [29]. This approach, along with anesthetic gas capture systems, eliminates the need for general anesthesia, reduces operating room time, decreases waste generation, and allows procedures to be performed in procedure rooms rather than operating rooms, yielding significant cost savings and carbon reductions [30,31].

Energy Efficiency Measures

Systematic energy management in operating rooms offers substantial opportunities to reduce carbon emissions and costs [32]. Energy efficiency measures include:

Optimizing HVAC systems: This is the highest-impact intervention, as HVAC accounts for over 90% of operating room energy use [3]. Strategies include adjusting ventilation rates based on occupancy and activity (reducing air changes per hour when rooms are unoccupied or during low-risk procedures), upgrading to energy-efficient systems, and implementing automated controls [3].

A multidisciplinary surgical team at Wrexham Maelor and Ysbyty Gwynedd Hospitals in the UK undertook a sustainable quality improvement project on carpal tunnel release surgery, gaining approval to perform procedures in procedure rooms rather than operating rooms, thereby enabling patients to bypass ward admission. The project forecast annual cost savings of £12,641 and carbon savings of 11.6 tonnes CO₂e per year (based on 75% applicability), equivalent to driving 33,285 miles in an average car [19].

Systemic upgrades: Upgrade to LED lighting, install motion sensors and timers, optimize equipment usage patterns, and ensure proper maintenance of all systems. New York-Presbyterian Hospital achieved annual savings of $1.77 million by replacing older lighting, air-conditioning, water-chilling, and pumping systems with more efficient models [7].

Sustainable Procurement and Circular Economy

A surgical waste audit of total knee arthroplasties revealed that a single procedure generates an average of 13.3 kg of waste, much of which consists of disposable linens and plastic packaging [32]. Consequently, a 2013 literature review found that reprocessing devices could reduce direct costs by 49% [33]. Life cycle assessments of reusable versus disposable surgical textiles and equipment consistently demonstrate environmental benefits for reusable products [34]. However, these benefits depend on efficient sterilization processes and sufficient product lifespan [35-37]. Specifically, a comparative life cycle assessment of laryngeal mask airways revealed that reusable versions have a significantly lower environmental footprint than disposable ones, provided they are not discarded prematurely [38] (Table 1).

**Table 1:** Key sustainability interventions and their environmental and economic benefits in perioperative care. This comprehensive table summarizes seven major intervention categories (Waste Management, Energy Efficiency, Anesthetic Gas Management, Sustainable Procurement, Digital Health, Clinical Optimization, and Education & Training) with specific interventions, quantified environmental benefits (waste reduction, carbon emissions, energy savings), economic benefits (cost savings, efficiency gains), and implementation frameworks aligned with the 5R principles and Green Team approaches. All interventions align with the UAE Net Zero 2050 mandate and the WHO sustainable healthcare guidelines. Source: Authors' own compilation based on systematic literature review of references.

Intervention category	Specific intervention	Environmental benefit	Economic benefit	Implementation framework
Waste management	Improved waste segregation, Blue wrap recycling, Reusable surgical gowns, Standardized surgical packs	50% reduction in medical waste volume over seven years [10], 66% reduction in GHG emissions [24], 50,000 lbs of waste prevented/year [10]	$60,000 annual savings [10], Reduced incineration costs	5R Framework: Reduce, Reuse, Recycle, Rethink, Research
Energy efficiency	HVAC optimization, LED lighting upgrades, Occupancy-based controls, Equipment maintenance	11.6 tonnes carbon emissions saved/year [19]	$1.77 million annual savings [10],£12,641 savings per facility [19], Reduced electricity costs	Green Teams + Systematic Energy Audits
Anesthetic gas management	Low-flow anesthesia (L/min), sevoflurane/isoflurane preference, Total intravenous anesthesia, WALANT technique	50–75% reduction in agent consumption [13], Up to 88% reduction in OR carbon footprint [20], Eliminates volatile gas emissions	Lower maintenance costs, Reduced anesthetic costs, Shorter OR times	Clinical Protocol Revision + Staff Training
Sustainable procurement	Reusable surgical instruments, Life-cycle assessments, Device reprocessing, Circular economy models	83% reduction in water use [24], 66% reduction in GHG [24], Lower environmental footprint	Cost-effective alternatives, 49% reduction in direct costs [33], Long-term cost savings	Supply Chain Redesign + Vendor Partnerships
Digital health	Telemedicine consultations, Electronic health records, Digital self-care platforms, Remote monitoring	58% fewer emissions vs. face-to-face [4], 66% reduction in travel-related emissions [4]	Reduced supply chain costs, Volume purchasing benefits, 300,000 electronic visits/year [9]	UAE Green Patient Care Project (EHS Model)
Clinical optimization	Minimally invasive surgery, Day-case surgery expansion, Getting It Right First Time, Regional anesthesia	26.5 kt reduction in carbon emissions [26], 380,000 inpatient bed days saved [4], Reduced resource intensity	Reduced facility overhead, Decreased patient travel costs, Increased efficiency	Evidence-Based Clinical Pathways
Education and Training	Staff sustainability training, Waste segregation education, Green Team workshops, Continuous professional dev.	Improved compliance rates, Better waste segregation, Increased awareness, Culture change	Reduced hospitalization costs, Improved patient outcomes, Reduced waste disposal costs	Workforce Development Programs

UAE context

The UAE has shown leadership in environmental sustainability by committing to achieving Net Zero emissions by 2050, becoming the first nation in the MENA region to set this ambitious target.

UAE National Net Zero 2050 Mandate

The UAE's Green Economy Strategy, launched in 2012, aims to position the nation as a global hub for green economy and sustainable development, with healthcare representing a critical sector for meeting these goals. The Net Zero 2050 Strategic Initiative is a comprehensive national commitment to carbon neutrality by mid-century, aligning with the Paris Agreement and positioning the UAE as a regional leader in climate action. The strategy covers all economic sectors, including healthcare, and requires systematic transformation of energy systems, industrial processes, transportation, and the built environment [6].

The healthcare sector's role in achieving Net Zero targets has been acknowledged but not fully operationalized. While the UAE has invested significantly in green building standards for new healthcare facilities and renewable energy infrastructure, the operational aspects of healthcare delivery, particularly perioperative care, have received less systematic attention. This presents both challenges and opportunities, as the high resource intensity of operating rooms means targeted interventions could yield substantial environmental benefits [6].

Current UAE Healthcare Sustainability Initiatives

The UAE healthcare sector has initiated several promising sustainability programs that provide a foundation for comprehensive perioperative sustainability efforts.

Emirates Health Services (EHS) launched the Green Patient Care project, leveraging artificial intelligence tools to estimate carbon emissions from in-person visits and using telemedicine to avoid them. In 2023, this initiative facilitated around 300,000 electronic visits, reducing CO₂ emissions associated with patient transportation by more than six million tons and raising patient awareness of their carbon footprint [9].

At the institutional level, Sheikh Shakhbout Medical City (SSMC) has emphasized that healthcare sustainability extends beyond green buildings to include safer, more efficient systems and supply chain management. Dr. Al Kaabi from SSMC highlights that "the greatest opportunities for reducing waste lie in safety practices and supply chain management," and that "even small, targeted improvements can make a big difference." This aligns with international evidence showing that operational improvements often bring greater sustainability benefits than infrastructure investments alone [10].

Additionally, the Abu Dhabi Public Health Center and the Partnership for Health System Sustainability and Resilience have highlighted the importance of embedding sustainability into health system planning and operations. Their 2025 report on Abu Dhabi's health system emphasizes the need for comprehensive approaches that consider environmental, economic, and social dimensions of sustainability [25].

Challenges in Waste Management 

Despite these advances, significant challenges remain in medical waste management across the UAE.

Healthcare waste management across the broader MENA region has historically faced infrastructure and operational barriers, as documented in studies from neighboring countries. A 2008 study by Al-Dahiri et al. found that not all UAE hospitals mark disposed containers or use waste tracking systems, and many lack pretreatment capabilities for medical waste-possibly due to budget constraints or the widespread use of disposable items [12].

Although incineration remains the predominant method of medical waste treatment, some facilities continue to operate outdated or poorly maintained incinerators, raising concerns regarding environmental safety. Furthermore, coordination among hospital departments remains suboptimal, limiting the effectiveness of waste management strategies [12].

More recent evidence reinforces these concerns. A 2021 assessment of Dubai Health Authority facilities found that the surveyed sites lacked waste-minimization practices, such as centralized purchasing and the selection of less-wasteful materials. Implementing these measures could significantly reduce waste volumes and the associated financial and environmental burdens [13].

Opportunities for Improvement and Future Directions

Addressing these gaps offers substantial potential for reducing both environmental impact and financial burden. Implementing targeted waste-reduction strategies--particularly in perioperative settings--could significantly reduce waste volumes and associated costs. These efforts are especially critical given rapid population growth and rising health care demand. Dubai’s population growth rate is projected to rise from 3.1% in 2018 to 4-5.5% by 2030, with additional pressure from expanding health tourism, further emphasizing the need for proactive sustainability planning [13].

At the same time, the UAE's strong commitment to innovation and technology adoption provides a unique opportunity to accelerate sustainable transformation. Investments in renewable energy, smart city infrastructure, and digital health systems create an enabling environment for implementing advanced sustainability solutions in healthcare. Building upon this technological foundation, the implementation of perioperative sustainability interventions must be prioritized based on regional feasibility and strategic alignment with the Net Zero 2050 mandate.

Digital health and telemedicine represent the highest-feasibility "low-hanging fruit," leveraging the UAE's world-class telecommunications infrastructure and existing EHS Green Patient Care frameworks to reduce travel-related emissions. Similarly, anesthetic gas transitions--specifically phasing out desflurane--require minimal capital investment and offer immediate carbon reductions through clinical protocol updates. However, from a resource-intensity perspective, energy-focused interventions, particularly HVAC optimization, likely offer the greatest regional impact. Given the UAE's extreme cooling requirements, occupancy-based airflow controls in operating theaters would yield a significantly higher carbon and financial return on investment (ROI) than in temperate climates.

Conversely, certain high-impact global strategies face regional hurdles; for instance, transitioning to reusable surgical textiles presents a moderate logistical challenge, requiring a strategic shift toward localized, water-efficient sterilization facilities to overcome current dependencies on global disposable supply chains. Nevertheless, international surveys indicate growing awareness and engagement among surgeons regarding sustainability in clinical practice, alongside increasing recognition of the need for systemic change [35]. By focusing on these high-impact, high-feasibility domains, the UAE is well-positioned not only to bridge the gap between aspirational policy and operational reality but also to influence broader sustainability practices across the MENA region.

## Conclusions

Aligning UAE perioperative care with the National Net Zero 2050 mandate is both an environmental imperative and an opportunity for healthcare system innovation. This review synthesizes global evidence showing that operating rooms contribute disproportionately to healthcare's environmental footprint through waste generation, energy consumption, and anesthetic gas emissions. The evidence base for perioperative sustainability is robust and actionable. Green teams implementing structured sustainability programs have achieved measurable reductions across multiple environmental domains while maintaining or improving patient safety and clinical outcomes. The 5R framework (Reduce, Reuse, Recycle, Rethink, Research) offers a systematic approach to identifying and implementing interventions, from waste segregation and reusable surgical textiles to anesthetic gas optimization and energy efficiency measures.

Critical gaps remain, notably the absence of UAE-specific perioperative sustainability data and the limited integration of healthcare into the National Net Zero 2050 roadmap. Addressing these gaps through systematic research, policy development, and stakeholder engagement could position the UAE as a regional leader in healthcare sustainability. The green scalpel is achievable through evidence-based interventions, multidisciplinary collaboration, and sustained commitment to environmental stewardship in surgical care.
